# Rapid Waterborne Pathogen Detection with Mobile Electronics

**DOI:** 10.3390/s17061348

**Published:** 2017-06-09

**Authors:** Tsung-Feng Wu, Yu-Chen Chen, Wei-Chung Wang, Ashwini S. Kucknoor, Che-Jen Lin, Yu-Hwa Lo, Chun-Wei Yao, Ian Lian

**Affiliations:** 1 VOR, Inc., San Diego 92122, CA, USA; winston@vorsense.com (Y.-C.C.); Johnnywang@vorsense.com (W.-C.W.); 2 Center for Advances in Water & Air Quality, Lamar University, Beaumont, TX 77710, USA; ashwini.kucknoor@lamar.edu (A.S.K.); Jerry.Lin@lamar.edu (C.-J.L.); cyao@lamar.edu (C.-W.Y.); 3 Department of Electrical and Computer Engineering, University of California at San Diego, San Diego, CA 92093, USA; ylo@ucsd.edu

**Keywords:** field testing, pathogen identification detection, mobile electronics, fluorescent-free labeling, waterborne

## Abstract

Pathogen detection in water samples, without complex and time consuming procedures such as fluorescent-labeling or culture-based incubation, is essential to public safety. We propose an immunoagglutination-based protocol together with the microfluidic device to quantify pathogen levels directly from water samples. Utilizing ubiquitous complementary metal–oxide–semiconductor (CMOS) imagers from mobile electronics, a low-cost and one-step reaction detection protocol is developed to enable field detection for waterborne pathogens. 10 mL of pathogen-containing water samples was processed using the developed protocol including filtration enrichment, immune-reaction detection and imaging processing. The limit of detection of 10 *E. coli* O157:H7 cells/10 mL has been demonstrated within 10 min of turnaround time. The protocol can readily be integrated into a mobile electronics such as smartphones for rapid and reproducible field detection of waterborne pathogens.

## 1. Introduction

Diseases caused by waterborne bacteria are common and lead to costly problems in public health. Data from the CDC shows that about 2.5 billion people worldwide, especially the populations in developing countries, suffer from a lack of quality drinking water [1,2,3,4]. For example, diarrhea, one of these waterborne diseases, leads to the death of over 800,000 children annually (more than AIDS, malaria, and measles) [1]. *Escherichia coli* (*E. coli*) is the one of major causative bacteria for waterborne diseases and commonly found in the intestines of animals and humans, which could be easily spread under inadequate hygiene or environmental control. In 2012, the U.S. EPA published a new guideline for recreational water pathogen control, defining >126 CFU of *E. coli* per 100 mL as unsafe to human health [5]. The standard protocol for the presence of *E. coli*, such as Method 1603 recommended by the EPA, requires a minimum of overnight culture to produce a detectable amount. The time-consuming bacterial culture cannot offer timely monitoring outcomes for water quality control. Further, in some conditions, it is shown that stressed *E. coli* cells might become viable but non-culturable while toxins are still secreted [6]. One major cause of waterborne outbreak is migration of bacteria within the circulatory pathway, especially from nonpoint source watersheds, where resources are limited for routine water quality monitoring. For instance, livestock on grazing lands are potential bacterial sources [7,8,9,10,11]. The bacteria from animal feces may leak into surface or underground water and eventually reach water facilities of human households. Hence, it is desirable to implement a simple and reliable field testing method to monitor waterborne bacteria such as *E. coli* on a more frequent basis. To develop such protocols, essential criteria including sufficient portability, ease of operation, short turnaround time, and high accuracy will need to be addressed [12,13,14,15,16]. Thus far, several potential biosensor technologies on mobile platforms for monitoring waterborne bacteria have been proposed [17,18,19,20,21,22,23,24,25]. However, some protocols require fluorescent labeling, which complicates sample preparation and discourage mobile platforms from the field testing of bacterial detection. To satisfy such challenges without significantly compromising sensitivity and specificity of detection, we propose a facile protocol including the following key features: (1) pre-enrichment of bacteria based on centrifugal membrane filtration; (2) quantitative detection of specific pathogen utilizing immunoagglutination reaction which is a one-step reaction and capable of achieving high selectivity and specificity without multiple washing steps [26,27]; and (3) a capillary driven microfluidic device, which transports introduced fluid sample without using external pump and also utilizes the narrow beam scanning technique compatible with the ubiquitous mobile electronics ideal for the purpose of waterborne field testing [28]. Due to the fact that *E. coli* O157:H7 can lead to serious human health issues such as bloody diarrhea, kidney failure, and even death, we have chosen this pathogen as the testing target and demonstrated the sensitivity and portability of our platform in this study [29,30,31].

## 2. Materials and Methods

### 2.1. Immunoagglutination Assays of Bacterial Detection

The anti-*E. coli* O157 latex microbeads were purchased from K&P Laboratories (Catalog# 5310-0346, Gaithersburg, MD, USA) and used without further modification. These antibody-coated microbeads (abMBs) are coated with polyclonal antibodies specifically against *E. coli* O157 for agglutination assays. In this study, heat-inactivated *E. coli* O157:H7 cells were purchased from K&P Laboratories (Catalog# 5370-0013, Gaithersburg, MD, USA), followed by dissolving with 1 mL of 1× phosphate-buffered saline. The number of *E. coli* O157:H7 cells was ~4 × 10^9^ per mL, which was calibrated by manual counting on the hemocytometer and consistent with data sheet from the vendor. Starting with the stock solution (5 × 10^9^ cells/mL), the serial dilution was operated in order to spike the designed E. coli cells including 1, 10, 10^2^, 10^3^, 10^4^ and 10^5^ cells into 10 mL of water samples for experiments. The solution of abMBs should be kept at room temperature for 10 min prior to use. For each experiment, 5 μL of abMB suspension (abMB concentration is ~9000 beads/μL, determined by using the hemocytometer) was added into enriched bacterium-containing samples. The mixed liquid samples of abMBs and *E. coli* O157:H7 cells were agitated with ultra-sonication for 2 min at room temperature, followed by their placement onto the microfluidic device. The specificity test was done by comparing bacterial detection with Salmonella, bought from K&P Laboratories (Catalog# 5370-0002, Gaithersburg, MD, USA). 

### 2.2. Membrane-Based Filtration for Pathogen Enrichment

The filtration process for water samples spiked with *E. coli* O157:H7 cells includes 2 steps: (1) coarse filtration: using a pore size of 40 μm cell strainer for pre-removal of unwanted impurities and (2) fine filtration: using an Amicon centrifugal filter with molecular weight cutoff (MWCO) of 100 kDa that has the membrane pore size of 30 nm to retain *E. coli* O157:H7 cells and remove excess water prior to introduction into the microfluidic device. In this study, we demonstrated the filtration process with an initial volume of 10 mL water samples. After the coarse filtration, the 10 mL of water sample was introduced into Amicon centrifugal filter, followed by centrifuge at 2000 g for 7 min. The final retentate volume was 50 μL, which was collected at the bottom of the centrifugal filter. The abMBs were introduced into the retentate in the centrifugal filter, where the immunoagglutination process occurred to form conjugates of *E. coli* cells and abMBs. 

### 2.3. Fabrication and Assembly of Microlens-Embedded Microfluidic Devices

**Figure 1:**
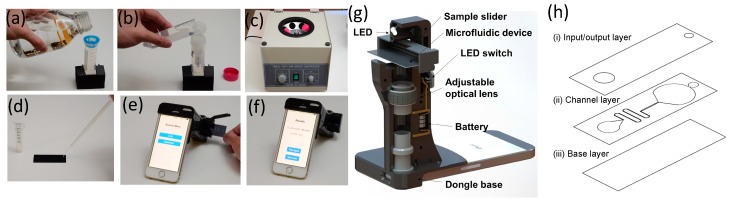
Flowchart of rapid field testing protocol for *E. coli* O157:H7 detection. (**a**) The 40 μm cell strainer is used for coarse filtration to remove unwanted large particles from 10 mL of containing *E. coli* O157:H7 cells-containing water samples; (**b**) Transfer the water sample into a centrifuge filter (pore size is ~30 nm) for further enrichment of pathogen samples; (**c**) Apply the centrifuge at 2000 g for 7 min, the resulting liquid volume is 50 μL. The antibody-coated microbeads are added into 50 μL of retentate in the centrifuge filter to initiate immunoagglutination reaction; (**d**) Dispense liquid samples containing conjugates onto a microlens-embedded microfluidic chip, where a narrow beam is formed to scan through the conjugates flowing within the microchannel (**e**,**f**) Insert a microfluidic device into a smartphone dongle and execute the smartphone application for bacterial counting (**g**) Schematic of the compact smartphone dongle for rapid waterborne field testing is illustrated, where the microfluidic device is inserted into dongle by using a sample slider. An LED powered by batteries is placed over the microfluidic device for illumination. The adjustable lens in the dongle tube is optically aligned with the CMOS camera on the smartphone; (**h**) The design of the capillary-driven microfluidic device includes (i) an input/output layer for sample introduction; (ii) a microchannel layer and (iii) a base layer.

The microlens used to produce the narrow beam for scattered light scanning was fabricated on the glass slide by a thermal reflowing process. The tilted narrow beam was used to produce scattered light from the agglutination. The detail of fabrication can be found in our previous study [28]. The design of microfluidic device is illustrated in Figure 1h, which includes an inlet, a curving microchannel, a viewing window as the sensing area, a waste collection reservoir and an outlet. The waste reservoir holds up to 25 μL of liquid. The microfluidic channel width at the entrance is 300 μm and expanded to 600 μm for the viewing window. The features of the microfluidic channel layer (thickness 50 μm) were made of a double-sided light blocking tape (FT 5250, Avery Dennison Corporation, Mentor, OH, USA) and prototyped by the razor cutter (Graphtec Inc., Irvine, CA, USA). As Figure 1h shows, the microchannel layer is bonded by the base layer and input/output layer both made of hydrophilic tapes with thickness of 100 μm (9962, 3 M, Maplewood, MN, USA). The finished microfluidic device is attached onto the glass slide with cylindrical microlenses fabricated on the opposite side of the glass slide. 

### 2.4. A Smartphone Dongle for Field Test Imaging

The smartphone dongle was used as a readout module. The module contains an LED for illumination, a sample slider, and an adjustable optical lens. It was prototyped using 3-D printer shown in Figure 1g. With proper optimization, this mechanical structure can be tailored for other mobile devices such as versatile Android devices as well. The dongle tube is optically aligned with the CMOS imager on an iPhone. The scattered light from abMBs flowing through the microchannel was recorded at a frame rate of 30 frames per second (fps) supported by CMOS imagers.

### 2.5. Protocol Operation

Figure 1a–f show an overview of the protocol used for enrichment and quantification of *E. coli* O157:H7 cells. 10 mL of a water sample containing bacteria was collected in a disposable container. A 40 μm cell strainer was placed on a 50-mL conical tube to filter out large impurities. The filtered water sample was then transferred to the Amicon centrifugal filter and centrifuged at 2000 g for 7 min. The retentate fluid volume after centrifugation was 50 μL. The abMBs was added into the retentate sample containing *E. coli* 157:H7 cells to initiate the immunoagglutination reaction. An exact volume micropipette was used to transfer 20 μL of immunoagglutination fluid sample from the centrifuge filter onto the inlet of the microfluidic device, where the channel made of hydrophilic tapes transported the fluid sample by capillary force through the sensing area. After inserting the microfluidic device into the smartphone dongle, users can start the iOS application for the automated bacterium quantification process. The overall protocol takes less than 10 min to bring the quantitative and specific bacterial test results.

### 2.6. iOS Application

**Figure 2:**
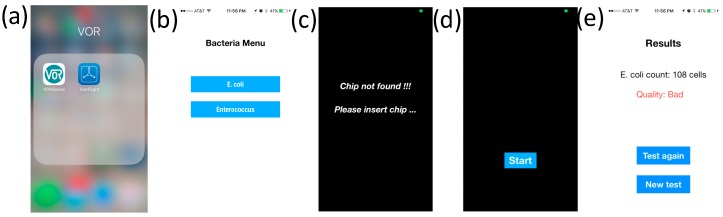
Screenshots of the *E. coli* software application execution on an iPhone. (**a**) Turn on the application by clicking the icon; (**b**) Choose *E. coli* on the window menu and an LED and CMOS camera of the smartphone are powered up; (**c**) Insert the sample slider, where the microfluidic device is placed, into the smartphone dongle. If the device is not detected, an error message will appear; (**d**) Start the image acquiring process by clicking the Process button on the screen. Both the dark-field scattering images and bright-field transmissive images are captured on the same CMOS camera; (**e**) Captured images are analyzed to determine the scattering intensity, which is used for the quantification of bacterium levels from 10 mL of water samples.

An iOS application was developed for readout analysis of scattered light on the same smartphone. The procedures are as follows and in Figure 2.

(a) Turn on the application by clicking the icon;
(b) Choose *E. coli* on the window menu and power up an LED and CMOS camera of the smartphone;
(c) Insert the sample slider (where the microfluidic device is placed) into the smartphone dongle;
(d) Start the image-acquiring process by clicking the Process button on the screen. Both dark-field scattering images and bright-field transmissive images are captured on the same CMOS camera;
(e) Captured images are analyzed to determine the scattering intensity, which is used for the quantification of bacterium levels.

## 3. Results

### 3.1. Scattered Light Detection by Narrow Beam Scanning Technique

**Figure 3:**
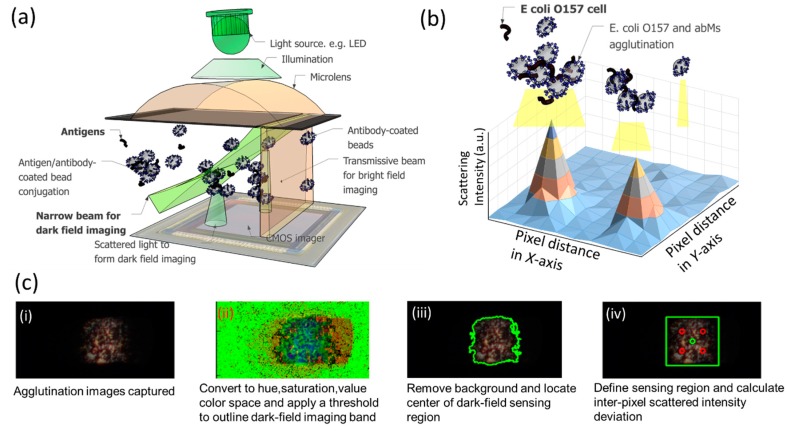
(**a**) Design of a narrow beam scanning microscopy compatible with the CMOS imager of mobile devices. The cylindrical microlens and the metal slit form a narrow beam of LED light, intersecting a moving bead and forming a dark-field image out of the large-angle (θ~40 degrees) scattered light. Also, the polymer lens material is autofluorescent, producing a weak light source to form a transmissive image of the microbead in a different region of the same CMOS imager. These closely correlated signals enable unambiguous determination of signals from the beads (which are proportional to bacterial counts) without the interference of dust particles or other debris in the sample; (**b**) The principal of analysing scattering intensity for quantitative *E. coli* detection. When introducing antibody-coated mircobeads into water sample containing *E. coli* cells, the immunoagglutination reaction forms different degree of agglutination. By using the narrow beam scanning technique, the scattered light from agglutination will be imaged on the smartphone CMOS imager. The size of agglutination depends on the levels of *E. coli* O157:H7 cells, resulting in the different patterns received. The image process algorithms can analyse the inter-pixel scattering intensity deviation to quantify pathogen levels; (**c**) The algorithms of image processing include (i) capture image by an iPhone CMOS camera (ii) convert the image into HSV (hue, saturation, value) color space and apply a threshold filter to outline the dark field imaging area; (iii) remove the background and locate the center of the imaging area; (iv) define the numbers of pixels for the sensing region to be processed and calculate deviation of scattered intensity within the defined sensing area.

The principle of this study for quantitative bacterial detection is to sense the scattered light from the conjugates of abMBs and *E. coli* O157:H7. The scatted light images captured on the CMOS imager enable the image processing to quantify levels of bacteria. As shown in Figure 3a, an incoherent light source (LED) illuminates abMBs by passing a cylindrical microlens made of photoresist. The photoresist-formed cylindrical microlens performs two functions: (a) light focusing as a regular lens to form dark-field images and (b) acting as a secondary light source to illuminate beads, forming bright-field images using the autofluorescence properties of the photoresist. The focused light is shaped into a narrow beam to produce large angle scattered signals, forming a dark-field image of conjugates of bacteria and abMBs. The secondary light source from the autofluorescence of the lens material forms a bright field image of the same conjugate in a different region of the same CMOS imager. These two correlated images, a dark-field large-angle scattering image and a bright-field transmissive image, enable us to exercise image processing algorithms to eliminate false signals due to dust particles or debris, greatly enhancing the sensitivity and accuracy of detection. Unlike other optical detection techniques which only collect the intensity change of signals, this narrow beam scanning technique can image the scattered light from abMB agglutination in the dark-field band. Utilizing the acquired images, we can clearly distinguish the levels of bacteria based on the inter-pixel standard deviation of scattered light. To determine the bacterial levels, 30 images are collected and analyzed for each sample. As shown in Figure 3b, it is expected that depending on the abundance of *E. coli* O157:H7 cells existing in the water sample, the agglutination degree will vary and thus the scattered light images captured will have different intensities on each pixel of the CMOS imager. As Figure 3c shows, the image-processing algorithm is applied to determine the inter-pixel standard deviation of scattered light within the defined processing area. The steps in this process are (1) The raw image is converted into HSV (Hue, Saturation, Value) color space, and a threshold filter is applied to remove the background noise; (2) The image is then converted to binary format, and topological structural analysis is applied to find the contours of the dark-field imaging area. We compress horizontal, vertical, and diagonal segments and leave only their end points to prevent heavy memory loading; (3) The contour describing the dark-field imaging area is computed using Green’s formula for defining the sensing region; (4) The inter-pixel standard deviation of the scattered light intensity within the processing area is returned. As *E. coli* O157:H7 levels in the water sample increase, the agglutinated clusters will produce a more concentrated scattering pattern and darken the other area, leading to a larger inter-pixel intensity standard deviation in the dark-field imaging band [32]. On the other hand, if an extremely low number of *E. coli* O157:H7 cells exist in the sample, minimum agglutination reaction will occur, and the dark-field imaging band will be entirely illuminated due to unbound abMBs, showing low inter-pixel intensity standard deviation. Compared to traditional methods that only measure intensity readout, our image-based algorithms could effectively avoid ambiguous situations such as detecting the same accumulative intensity produced by a similar amount of abMBs, regardless of the actual number of bacteria. Also, since the narrow beam scanning creates extremely bright spots in the dark-field band, the image quality on the smartphone CMOS camera is expected to achieve a high signal-to-noise ratio for detection.

### 3.2. Agglutination Reaction Optimization

After the filtration process, 99.5% of liquid is removed from the original water sample, yielding a 200-fold enrichment in sample concentration and accelerated immunochemistry reaction rate. Traditionally, immunoagglutination is only used in a qualitative test; however, together with the narrow beam technique, immunoagglutination offers a simple, generally applicable, and non-hazardous method for fast and bacterium-specific quantitative detection [33,34,35,36,37,38,39,40,41,42,43,44,45,46]. More importantly, the agglutination reaction is a one-step reaction method mediated by specific reactions between antibodies immobilized on microbeads and antigens in the sample, requiring no further washing steps prior to detection. This one-step reaction ensures that users can implement field testing in the presence of bacteria anytime. The critical factor in achieving high detection performance is the optimal ratio of antibody to antigen to avoid an agglutination reaction in the antigen excess zone under the Heidelberger-Kendall curve [18,19]. It is understood that as the concentration of bacteria increases beyond the optimal detection region, fixed amount of abMBs are unable to capture all increased *E. coli* O157:H7 cells, and the binding sites of antibodies on microbeads are saturated. Therefore, few *E. coli* O157:H7 cells will be shared with two or more abMBs, resulting in minimal agglutination clusters. The optimal reaction region can only be defined empirically for different detection purposes. In our protocol, the total number of abMBs introduced to each detection is calibrated to around 45,000 beads to implement the dynamic range from 1 to 10^5^ *E. coli* O157:H7 cells for given antibody used. 

### 3.3. Capillary Driven Microfluidic Device Fabrication

To execute field testing for pathogen detection, the capillary-driven microfluidic device was applied in this study without any external pumps. When dispensing a fluid sample containing *E. coli* O157:H7 into the inlet, the capillary effect from hydrophilic tapes drove liquid through the microfluidic channel. The *E. coli* O157:H7/abMBs agglutinated cluster in the fluid sample flowed through the downstream channel for optical scanning. Due to the coarse filtration step, there is no large dust particles or impurities that can cause clogging issues in the microfluidic channel. The size of an agglutinated cluster is around 20–40 μm, which can be accommodated by the microchannel geometry (300 μm (w) × 50 μm (h)) without blocking the microchannel or slowing down the flow. The optimization of the microchannel width is important to observe the change of scattered light from agglutination. For the channel width less than 300 μm, given the introduced abMB numbers, the overlapping effect of abMBs in the microchannel results in less sensitive detection performance for the low levels of *E. coli* O157:H7 from 10 to 10^3^ cells. Because less agglutination occurs in the environment with fewer *E. coli* cells, most abMBs are monodisperse and overwhelm the entire microchannel, making inter-pixel deviation change of scattered light invisible. Further, the narrower channel width also leads to a faster flow rate, resulting in the incapability of the smartphone CMOS camera to record distinguishable images. 

**Figure 4:**
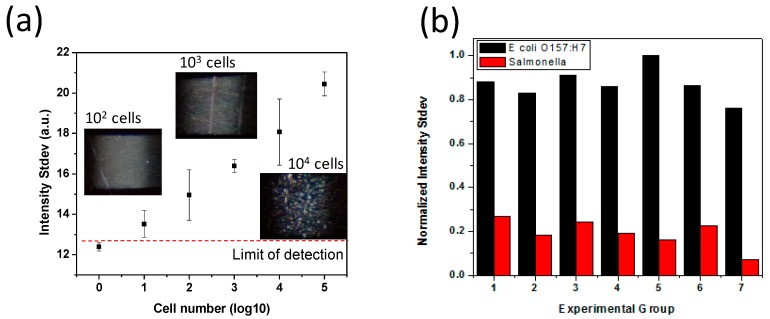
(**a**) Standard curve of *E. coli* O157:H7 spiked into 10 mL of water by using pathogen detection protocol. Each *E. coli* O157:H7 level was examined in three different experiments. The detection limit of the system is shown to below 10 cells; (**b**) Specificity test of *E. coli* O157:H7 versus *Salmonella*, wherein the narrow beam scanning technique shows clear differentiation from Salmonella.

Figure 4a shows the standard curves for *E. coli* O157:H7, which were generated using a series of dilutions of standard *E. coli* O157:H7 cell suspension between ~5 cell and ~5 × 10^5^ cells in 10 mL of water. The linearity between pixel standard deviation and the number of spiked cells is observed over 5 orders of magnitude. The limit of detection (LOD) in the 10-mL sample, defined by the capability of discerning the measured number from the 3-sigma of the lowest *E. coli* levels detected in this study, is calculated to be around 10 cells. Together with the LOD, this study provides an appropriate detection range for field testing needs since the infectious dose of 10-100 *E. coli* O157:H7 cells is considered harmful for human health. Even though the cross-reactivity of *E. coli* O157:H7 detection is mainly determined by the antibody, the specificity of *E. coli* O157:H7 versus inactivated Salmonella cells (purchased from K&P Laboratories, Gaithersburg, MD, USA) was examined by using the narrow beam scanning technique. Figure 4b shows the discernible cutoff threshold to separate *E. coli* O157:H7 from Salmonella at 5 × 10^4^ cells, indicating this protocol offers a specific method for rapid pathogen detection.

We next evaluated the recovery rate when water samples containing *E. coli* O157:H7 cells flowed through coarse filtration and centrifuge filters. Since the retentate volume after the filtration process is 50 μL, the same amount of *E. coli* O157:H7 cells were spiked into each 50 μL sample of control water. Without executing the centrifuge process, we should expect the control samples to represent a 100% recovery rate. Triplicate experiments implemented on each control condition were compared to the experiment using the proposed protocols. The recovery rate is estimated to be about 90%, which is acceptable when applying liquid transfer in this study.

**Figure 5:**
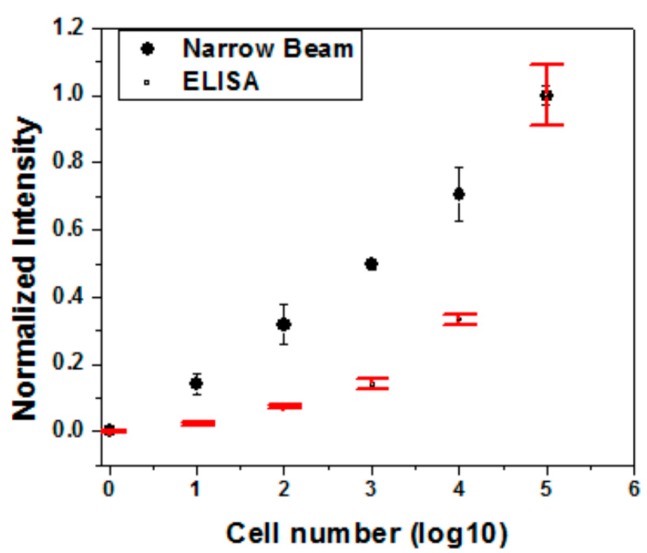
Titration experiments with water samples that were spiked with different levels of *E. coli* O157:H7 cells. The narrow beam scanning technique shows better linearity within the dynamic range of interest. All experiments were performed in triplicates. The graphs show as mean ± standard deviation.

We also benchmarked the detection performance of the proposed protocol with the enzyme-linked immunosorbent assay (ELISA) method. The MaxSignal *E. coli* O157:H7 test kit (Bio Scientific Corp., Austin, TX, USA) was utilized following the manufacturer’s instructions. The microwells were measured at 450 nm wavelength by the ELISA reader (EL × 800, BioTek inc., Winooski, VT, USA). The optical densities of the samples were determined and compared with that of the kit standards. The same *E. coli* O157:H7 dilutions from 5 cell to 5 × 10^5^ cells were separately measured with both the narrow beam scanning technique and the ELISA method. In Figure 5, both measurements show a consistent readout to distinguish *E. coli* O157:H7 levels. However, the detection outcomes from the narrow beam scanning platform show better linearity than the ELISA method within the dynamic range of interest. 

## 4. Conclusions

We have demonstrated a novel narrow beam scanning technique implemented on smartphone platforms as a field-deployable detection method for waterborne pathogens. Without any washing steps, this approach applies a one-step immunoagglutination reaction for quantitative pathogen detection. The narrow beam scanning technique allows for the execution of imaging process algorithms to examine inter-pixel scattering intensity deviation in dark-field imaging bands. Since the scattered images are produced from the agglutinated cluster of antibody-coated microbeads and pathogens, this protocol provides a pathogen-specific and high-accuracy detection outcome. Even though the platform was specifically validated on the *E. coli* O157:H7 and successfully achieved the desirable detection limit of quantifying 10 cells from 10 mL of water sample, the proposed protocol can be facilely expanded on other bacterial detection for waterborne pathogen monitoring and outbreak control. In addition, the enrichment protocol by centrifugal membrane filtration can be replaced with other membrane filtration methods such as the vacuum method to further simplify deployment of the field testing. The microfluidic chip can be further tailored for multiplex pathogen detection by immobilizing two or more antibodies on the chip substrate to capture pathogens in the sample. The pathogen-containing sample is then introduced into microfluidic chips together with the detecting-antibody-coated microbeads. This integrated protocol is simple and capable of delivering the result within 10 min, representing a major advantage over the currently overnight, culture-based bacterial tests.
